# Effects of Prenatal Exposure to Ozone, Heatwave and Green Space on Neonatal Congenital Heart Disease: A Case-Control Study in Eastern China

**DOI:** 10.3390/toxics13090716

**Published:** 2025-08-26

**Authors:** Weizhe Zhang, Tiezheng Li, Leiyu Shi, Die Li, Mary A. Fox

**Affiliations:** 1 Health Policy & Management, Johns Hopkins Bloomberg School of Public Health, Baltimore, MD 21202, USA; wzhang94@jh.edu (W.Z.); lshi2@jhu.edu (L.S.); 2 Innovation Center for Child Health, Binjiang Institute of Zhejiang University, Hangzhou 310000, China; 12018347@zju.edu.cn; 3 Heart Center, National Clinical Research Center for Child Health, Children’s Hospital, Zhejiang University School of Medicine, Hangzhou 310000, China; 4 Health Policy & Management and Environmental Health & Engineering (Joint), Johns Hopkins Bloomberg School of Public Health, Baltimore, MD 21202, USA

**Keywords:** congenital heart disease, maternal exposure, ozone, green space, heatwave

## Abstract

Congenital heart disease (CHD) is the most prevalent birth defect. Ozone and heatwave exposure during pregnancy could increase the risk of adverse birth outcomes. Green space might be associated with beneficial birth outcomes. The research on the combined effects of those exposures on CHD is limited. Therefore, we conducted a multicenter case–control study based on a surveillance system in Zhejiang Province, China, to explore the effect of ozone, heatwave, and green space exposure during early pregnancy on CHD and their interaction. The inverse distance weighting method and normalized difference vegetation index were applied to assess maternal ozone and green space exposure, respectively. The heatwave definition is from the National Oceanic and Atmospheric Administration. Our study reveals positive associations of heatwave and ozone exposure with CHD (ozone: OR = 1.07, 95% CI: 1.02, 1.13; heatwave: OR = 1.29, 95% CI: 1.18, 1.40), and green space in different buffers around residence exerted protective effects on CHD, with ORs ranging from 0.93 to 0.94. Associations between ozone and CHD were weakened among participants with higher NDVI. Ozone’s effects on CHD were stronger with the increased duration of heatwave exposure. Our study indicates that ozone and heatwave exposure could increase the risk of CHD, and high green space is a protective factor for CHD. Meanwhile, high green space exposure could attenuate the effect of ozone on CHD, but heatwave exposure strengthened it.

## 1. Introduction

Congenital heart disease (CHD), as the most prevalent birth defect, accounted for 261,247 infant deaths globally in 2017 [1]. CHD refers to congenital malformations caused by abnormal heart and major blood vessels development during the embryonic period. Although genetic factors are dominant in the pathology of CHD, the effect of environmental factors has been highlighted with the change in lifestyle and climate in recent decades [2,3,4].

Numerous studies have revealed that air pollutants, such as ozone (O_3_), particulate matter ≤ 5 μm (PM_2.5_), particulate matter ≤ 10 μm (PM_10_), nitrogen dioxide (NO_2_), and carbon monoxide (CO), are significant contributors to CHD and other birth defects. For example, a systematic review suggested positive associations between maternal exposure to O_3_, PM_2.5_, PM_10_, NO, and CO and the risk of specific CHD subtypes, including atrial septal defect (ASD) and tetralogy of Fallot (TOF) [5]. Meanwhile, air pollutants were also reported to elevate the risk of other adverse birth outcomes, such as preterm birth, low birth weight, and cleft palate [6,7,8]. Although the concentration of air pollutants, such as PM_2.5_, PM_10_, and NO_2_, is decreasing due to air pollution control policies, ozone concentration has been increasing worldwide due to global warming in recent years [9]. The impact of ozone on CHD has attracted increasing attention. A multicenter study showed that an increment of 10 µg/m^3^ in ozone exposure during early gestation was positively associated with CHD [10]. A nested case–control study reported a positive association between CHD and ozone exposure during the first week of pregnancy [11]. However, a systematic review did not observe any significant association between ozone and CHD after performing a meta-analysis on 32 studies [12].

Rapid urbanization has reduced the contact between urban residents and the natural environment in recent decades. Green spaces around residents have become the most direct and effective way to contact nature. Numerous studies have verified that high green spaces exposure could lower the risk of obesity, diabetes, tumors, neurodegenerative disease, and overall mortality [13,14,15,16,17,18]. Meanwhile, green space exposure during pregnancy was associated with a lower risk of adverse birth outcomes such as premature birth and low birth weight [19]. The protective mechanism of residential green spaces on health is still unclear. However, researchers speculated that green spaces might reduce environmental health-damaging factors, such as air pollution, noise, and heat [20,21,22]. In addition, green space can provide an ideal place for physical exercise and interpersonal communication, thereby promoting social interaction, relieving psychological stress, preventing depression, and maintaining people’s physical and mental health [23]. Although increasing studies have focused on the role of residential green space on congenital defects, the evidence of green space’s effect on CHD is relatively limited.

In the context of climate change, the global temperature has increased by 1.25 °C due to human activities in recent decades and will exceed 1.5 °C in 10 years [24]. The rising temperature has aggravated the magnitude, frequency, and duration of extreme weather events, such as heatwaves, threatening human health. As one of the most hazardous environmental factors, extreme heat exposure led to 489,000 excess deaths annually from 2000 to 2019 [25]. Compared with the normal population, pregnant women are more vulnerable to external heat, since their thermoregulation systems will be challenged by the physiological and anatomical changes during pregnancy [26]. Previous studies indicate that increased temperature or heatwave exposure during pregnancy might result in higher risks of adverse birth outcomes, including preterm birth, stillbirth, and low birth weight [27,28]. A large population-based case–control study reported that extreme heat exposure during pregnancy could increase the risk of ventricular septal defect (VSD) and atrial septal defects (ASD) [29]. Xu et al. also found that increased weekly temperature exposure was positively associated with elevated risk of CHD [30]. Studies have reported that heat exposure could boost the effect of air pollutants on human health [31,32], but the combined effect of heatwaves and ozone on CHD still needs to be explored.

To elucidate the effects of ozone, green space, and heatwave during pregnancy and their combined impact on CHD, we conducted a case–control study based on a neonatal congenital heart disease surveillance system covering Zhejiang, a province located along the southeast coast of China. Besides exploring the associations of ozone, green space, and heatwave exposure during early pregnancy with CHD, we also estimated the interactive effect of that exposure. Our study will provide evidence to verify the impact of multiple maternal exposures on infant health and help improve health policy and urban planning.

## 2. Methods

### 2.1. Study Population

We selected the study population from infants born from January 2019 to June 2022 in medical institutions in Zhejiang Province and their mothers. The infant and maternal information, such as maternal age, gestational weeks, infant sex, and birth weight, was derived from the Network Platform for Congenital Heart Disease (NPCHD). The NPCHD was established in 2012 under the cooperation of major medical institutions in Zhejiang Province, aiming to provide early screening, diagnosis, management, and treatment for newborns with CHD, supporting the prevention and control of CHD. In 2023, the NPCHD covered 11 cities and more than 95% of newborns in Zhejiang Province. In the routine medical process, the basic information of each newborn and their mothers will be uploaded to the NPCHD, along with the infant’s diagnosis data. In principle, each child born in Zhejiang Province should be screened for CHD by percutaneous arterial oxygen saturation (POX) test and cardiac auscultation within three days after birth. Children who are suspected of being positive will be further examined by echocardiogram. In this study, we identified 8307 CHD cases according to the diagnosis information in the NPCHD from January 2019 to June 2022. We excluded infants without detailed birth diagnosis information and pregnant women without complete information on covariates. Because we assessed the maternal exposure to the heatwave and green space according to the residential address, pregnant women who did not reside in Zhejiang Province before delivery or who did not have detailed addresses were also excluded. Meanwhile, we matched infants without CHD or other birth defects and their mothers by maternal age on a ratio of 1:3. In total, 6809 CHD infants and their mothers were included in the case group, and 20,427 healthy infants and their mothers were included in the control group. The detailed procedure for selecting the study population is shown in Figure S1.

### 2.2. Covariates

The baseline information of infants and their mothers was obtained from the NPCHD, including maternal age, gestational weeks, infant sex, birth weight, primipara or multipara, singleton or multiple pregnancy, conception season (spring, summer, autumn, or winter), and residential region (urban or rural). The maternal age was calculated by subtracting the birthday from the delivery date. The gestational weeks are calculated based on the first day of the last menstrual period, and there is a gestational week every seven days thereafter. The conception seasons were defined according to the Chinese climate characteristics: spring (1 March to 31 May), summer (1 June to 31 August), autumn (1 September to 30 November), and winter (1 December to 28 February). We also collected the average years of education per capita and per capita disposable income from the statistical communique released by the county-level administrative district to measure the economic development level of the areas where participants resided [33]. In this study, the areas where per capita disposable income is lower than the average per capita disposable income of Zhejiang Province were defined as low-income areas; otherwise, the areas were designated as high-income areas.

### 2.3. Ozone Exposure Assessment

The daily average ozone concentration monitoring data were obtained from the Qingyue Open Environmental Data Center (http://data.epmap.org/). Using the Amap API Server, we converted each monitoring site’s location and each participant’s residential address to WGS-84 coordinates. We applied the inverse distance weighting (IDW) method [34] to assess the average ozone concentrations between 3 and 8 weeks after conception, the cardiac critical period. The calculation formula of the inverse distance weighting is:

(1) $$W_{i}=\;\frac{d_{i}^{-p}}{\sum_{i=1}^{n}d_{i}^{-p}}$$

$W_{i}$ is the weight of the distance from the pregnant woman’s residential address to each monitoring station; $d_{i}$ is the distance from participants’ residences to air pollution monitoring points; *p* is the power parameter, which is an arbitrary positive number and usually takes a value of 2 [35]. The calculation formula of ozone concentrations for each participant is:

(2) $$\hat{Z}(X_{0},Y_{0})=\;\sum_{i=1}^{n}W_{i}Z(X_{i},Y_{i})$$

$\hat{Z}(X_{0},Y_{0})$ and $Z(X_{i},Y_{i})$ are the ozone concentrations of participants and monitoring sites, respectively.

### 2.4. Green Space Assessment

We used the normalized difference vegetation index (NDVI), an indicator for the density of green vegetation on the ground, to assess the residential greenness of each participant [36]. The calculation formula is:

(3) $$NDVI=\;\frac{NIR-R}{NIR+R}$$

*NIR* is the reflection value of the near-infrared band, and *R* is the reflection value of the red-light band. A positive NDVI value indicates a green vegetation covered area. We calculated the average NDVI in 500 m, 1000 m, and 1500 m circular buffers around participants’ addresses between 3 and 8 weeks after conception to indicate green space exposure. The multiband images we used were captured by the Sentinel-2 satellite with 50 m spatial resolution and 14-day temporal resolution. Cloud cover is an important factor affecting the image quality, and we remove the cloud in images during the preprocessing stage, resulting in void areas. Therefore, we used images with exact spatial and temporal resolutions captured by the Landsat-8 satellite to fill void areas. Sentinel-2 and Landsat-8 imagery was obtained via Google Earth Engine (GEE, https://earthengine.google.com/).

### 2.5. Heatwave Identification

Since there is no universal heatwave definition, we applied the NOAA definition, from the National Oceanic and Atmospheric Administration (NOAA), where the heat index (HI) exceeds a specific threshold for several days. The HI is an index that considers air temperature and relative humidity to determine the temperature the human body feels, also known as the perceived temperature. Compared to the absolute temperature, the HI could better reflect the actual feeling of the human body in a high-temperature and high-humidity environment. To adapt to local climate characteristics, we calculated the mean heat index within the warm seasons (1 May to 30 September) in the 5 years before conception as the threshold for each pregnant woman. In this study, NOAA_1 day, NOAA_2 days, and NOAA_3 days denote the daily heat index exceeding the mean heat index by 10 degrees and lasting 1, 2, and 3 consecutive days, respectively. We calculated participants’ heatwave exposure based on their address in Google Earth Engine (GEE) with R software (version 4.3.3). The temperature data was derived from the European Centre for Medium-Range Weather Forecasts (ECMWF) ERA5 Land Reanalysis dataset. The ERA5 dataset has been widely validated against ground-based meteorological station observations in China, including in eastern coastal provinces such as Zhejiang [37]. Previous studies reported high consistency, with correlation coefficients (R^2^) exceeding 0.90 and root mean square errors (RMSE) typically between 0.95 °C and 3.11 °C [38]. The heat index was computed based on the hourly air temperature, saturation vapor pressure, and partial water vapor pressure. We calculated the HI using the Rothfusz regression adopted by the NOAA National Weather Service:

HI = −42.379 + 2.04901523*T* + 10.14333127*R* − 0.22475541*TR* − 6.83783 × 10^−3^*T*^2^ − 5.481717 × 10^−2^*R*^2^ + 1.22874 × 10^−3^*T*^2^*R* + 8.5282 × 10^−4^*TR*^2^ − 1.99 × 10^−6^*T*^2^*R*^2^ (4)

where *T* is the ambient air temperature and *R* is the relative humidity in percent.

(5) $$R=\frac{E}{E_{s}}\;\times \;100\%$$

(6) $$E=E_{0}\times \;exp\left(\frac{L}{R_{v}}\times \left(\frac{1}{T_{0}}-\frac{1}{T_{d}}\right)\right)$$

(7) $$E_{s}=E_{0}\times \;exp\left(\frac{L}{R_{v}}\times \left(\frac{1}{T_{0}}-\frac{1}{T_{a}}\right)\right)$$

where $E_{0}$ = 0.611 is the saturation vapor pressure at a reference temperature $T_{0}$ = 273 Kelvin (K). L = 2.5 × 10^6^ J kg^−1^ is the latent heat of evaporation for water, and $R_{v}$ = 461.52 J kg^−1^ K^−1^ is the specific gas constant for water vapor. $E_{s}$ is the partial water vapor pressure at given dew point temperature $T_{d}$ in Kelvin. $E_{s}$ is the saturation vapor pressure at given air temperature $T_{a}$ in Kelvin.

### 2.6. Statistical Analysis

Categorical variables, such as infant sex, primipara or multipara, and conception season, were presented as percentages. Normally distributed variables, such as maternal age, gestational weeks, and birth weight, were presented as mean ± standard deviation (SD). Skewed distribution variables, such as the NDVI, were presented as median and interquartile range (P_25_, P_75_). The Chi-square test, Student’s *t*-test, and Wilcoxon signed-rank test were used to test the difference between groups of categorical variables, normally distributed variables, and skewed distribution variables, respectively.

The associations between ozone exposure, green space, heatwave, and the risk of CHD were evaluated by the multivariate logistic regression model, which was progressively adjusted for covariates, including infant sex, mother’s age, birth weight, gestational weeks, conception seasons, parity (primipara or multipara), singleton or multiple pregnancy, residential region (urban or rural) and per capita disposable income. Restricted cubic splines were drawn to present the exposure–response curves between ozone concentration, NDVI, and the risk of CHD. Besides the overall CHD, we also explored the associations of heatwave, NDVI with common CHD subtypes. We explored the interactive effects between ozone and green space on the risk of CHD by including interaction terms (ozone × NDVI) in logistic regression models and estimating the odds ratios (ORs) for the association between ozone exposure and CHD across different quartiles of NDVI. Similarly, interaction terms between ozone and heatwave were incorporated into the models to evaluate their joint effects, and stratified analyses were performed across varying levels of heatwave exposure. *p*-values for interaction were calculated to assess the statistical significance of the observed effect modifications. Subgroup analyses were performed according to infant sex (boy or girl), maternal age (<35 or ≥35 years), residential region (urban or rural), and per capita disposable income (low or high). Several sensitivity analyses were performed to strengthen our results: (1) excluding infants whose birth weight was <2.5 kg; (2) excluding mothers whose gestational week was <37 weeks. (3) excluding multiple pregnancy.

All statistical analyses were performed using R software (version 4.5.1). Appropriate packages and functions were applied for regression modeling, interaction analyses, and visualization as described in the relevant sections.

## 3. Results

### 3.1. Baseline Characteristics of the Study Population

Table 1 shows the characteristics of the study population. In all participants, the average age of pregnant women was 30 ± 5 years, and the percentages of male and female infants were 47.40% and 52.60%, respectively. The average gestational weeks of CHD cases were longer than those of controls. The case groups had higher average birth weight, primipara, and singleton pregnancy percentages than the control group. The case group had a higher percentage of participants who lived in urban, high-education and high-income areas. The case group experienced higher ozone exposure and longer heatwave exposure duration (2 days and 3 days) than the control group. The NDVI in 500 m, 1000 m, and 1500 m buffers were higher in the control group.

### 3.2. Maternal Ozone Exposure and CHD

**Figure 1:**
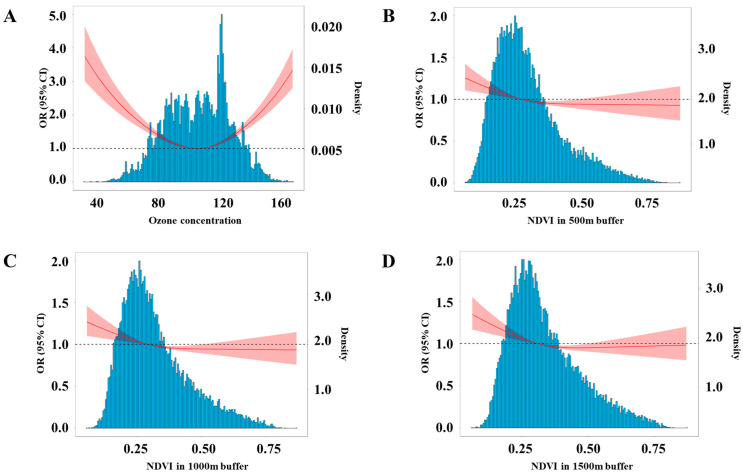
Exposure–response curves between maternal exposure and the risk of CHD in offspring. The blue bars represent population density. The red line indicates the OR values, and the dashed line represents OR = 1. (**A**) Exposure–response curves between ozone exposure and CHD. (**B**) Exposure–response curves between green space exposure in 500 m buffer around residence and CHD. (**C**) Exposure–response curves between green space exposure in 1000 m buffer around residence and CHD. (**D**) Exposure–response curves between green space exposure in 1500 m buffer around residence and CHD. Each model was adjusted for infant sex, mother’s age, birth weight, gestational weeks, conception seasons (spring, autumn, fall, and winter), parity (primipara or multipara), singleton or multiple pregnancy, residential region (urban or rural), average years of education per capita, and per capita disposable income (low and high).

The odd ratios (ORs) and 95% confidence interval (95% CI) for associations between maternal exposure and CHD are presented in Table 2. We divided the study population into four groups according to the quartiles of ozone concentration and NDVI, respectively. The lowest groups (Q1) were designated as reference groups to evaluate associations of ozone and NDVI with CHD. We found that ozone exposure elevated the risk of CHD (OR = 1.07, 95% CI: 1.02, 1.13 per IQR increment). Meanwhile, compared to the Q1 group, ozone presented a protective effect on CHD in the Q2 group (OR = 0.83, 95% CI: 0.76, 0.90) but exerted an adverse effect on CHD in the highest group (Q4: OR = 1.40, 95% CI: 1.28, 1.54). Therefore, we speculated that ozone and CHD might have a U-shaped association. The restricted cubic spline also showed the U-shaped exposure–response curve association between ozone and CHD (Figure 1A).

### 3.3. Maternal Green Space Exposure and CHD

We hypothesized that green space could reduce the risk of CHD. Each interquartile range (IQR) increment in NDVI in 500 m, 1000 m, and 1500 m buffer around participants’ residence lowered risk of CHD by 6%, 5%, and 6%, respectively (NDVI in 500 m buffer: OR = 0.93, 95% CI: 0.90, 0.97; 1000 m buffer: OR = 0.94, 95% CI: 0.90, 0.98; 1500 m buffer: OR = 0.93, 95% CI: 0.89, 0.97). Figure 1B–D show the exposure–response curves of NDVI in 500 m, 1000 m, and 1500 m buffers with CHD. The risk of CHD is reduced with the increase of NDVI in 500 m and 1500 m buffers.

### 3.4. Maternal Heatwave Exposure and CHD

As for the heatwave exposure, we assigned the participants not exposed to heatwave as reference groups to estimate the association between heatwave and CHD. Heatwave exposures lasting 2 days or 3 days were risk factors for CHD (NOAA_2 days: OR = 1.31, 95% CI: 1.19, 1.44; NOAA_3 days: OR = 1.29, 95% CI: 1.18, 1.40).

### 3.5. Sensitivity Analyses

Sensitivity analyses show that the associations of heatwave and green space with CHD were consistent with the main analyses after excluding infants whose birth weight was lower than 2.5 kg, mothers whose gestational week was lower than 37 weeks, and those who had multiple pregnancies (Table S1).

### 3.6. Subgroup Analyses

Tables S2–S5 show the results of subgroup analyses. All the *p* values for interaction were over 0.05, indicating that the associations of maternal ozone, green space, and heatwave exposure with CHD did not vary across infant sex (Table S2) and maternal age (Table S3). After stratifying associations by region (Table S4) and income (Table S5), we found that participants who lived in rural regions benefited more from the green space in 1000 m and 1500 m buffer than those who lived in urban areas. Moreover, mothers who lived in urban and high-income areas were more susceptible to heatwave exposure (*p*_for interaction_ < 0.05). The associations between ozone and CHD did not vary across residential regions and income levels (*p*_for interaction_ > 0.05).

### 3.7. Maternal Exposures and CHD Subtypes

Table 3 shows the associations of maternal exposure with common CHD subtypes. Overall, the effects of ozone, green space, and heatwave on CHD subtypes were inconsistent. We observed ozone exposure could elevate the risk of VSD, AVSD, and PDA (VSD: OR = 1.20, 95% CI: 1.06, 1.36; AVSD: OR = 1.29, 95% CI: 1.15, 1.45; PDA: OR = 1.26, 95% CI: 1.17, 1.36), but there was no association between ozone and ASD (OR = 0.97, 95% CI: 0.91, 1.04). Moreover, we found the protective effects of green space on ASD and PDA. Although the longest duration of heatwave (3 days) exposure could increase the risk of all subtypes, the heatwave lasting 1 day and 2 days exerted different effects on CHD subtypes. For example, heatwave lasting 1 day was a protective factor for ASD (OR = 0.72, 95% CI: 0.64, 0.80) but a risk factor for AVSD (OR = 1.28, 95% CI: 1.09, 1.51), heatwave lasting 2 days had positive associations with ASD and AVSD (ASD: OR = 1.39, 95% CI: 1.24, 1.56; AVSD: OR = 1.54, 95% CI: 1.27, 1.85), but had no significant association with VSD and PDA (VSD: OR = 0.95, 95% CI: 0.75, 1.19; PDA: OR = 1.07, 95% CI: 0.94, 1.21).

### 3.8. Interaction Between Ozone, Green Space and Heatwave

**Figure 2:**
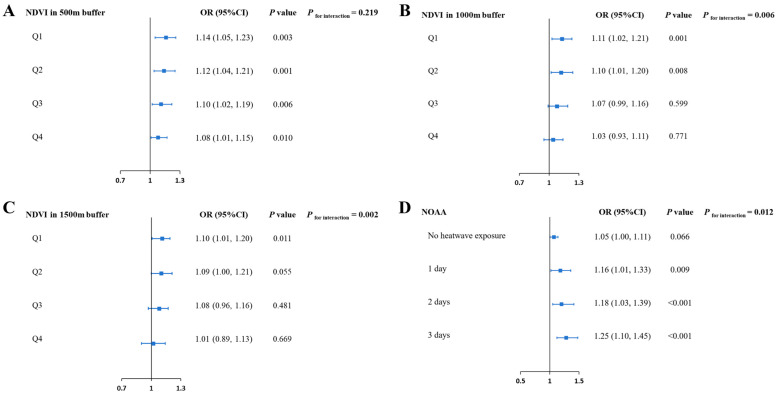
Associations between maternal ozone exposure and CHD in different levels of green space and heatwaves. (**A**) Associations between maternal ozone exposure (per IQR increment) and CHD in different quartiles of NDVI in 500 m buffer; (**B**) associations between maternal ozone exposure (per IQR increment) and CHD in different quartiles of NDVI in 1000 m buffer; (**C**) associations between maternal ozone exposure (per IQR increment) and CHD in different quartiles of NDVI in 1500 m buffer; (**D**) associations between maternal ozone exposure and CHD in different levels of heatwaves; each model was adjusted for infant sex, mother’s age, birth weight, gestational weeks, conception seasons (spring, autumn, fall, and winter), parity (primipara or multipara), singleton or multiple pregnancy, residential region (urban or rural), average years of education per capita, and per capita disposable income (low and high).

Figure 2 presents the associations between ozone and CHD in different levels of NDVI and heatwave. The associations between ozone and CHD were significant in all quartiles of NDVI in a 500 m buffer (Figure 2A). However, statistically significant associations were not found for higher levels of NDVI in 1000 m buffer (Q3: OR = 1.07, 95% CI: 0.99, 1.16; Q4: OR = 1.03, 95% CI: 0.93 1.11) and 1500 m buffer (Q3: OR = 1.08, 95% CI: 0.96, 1.16; Q4: OR = 1.01, 95% CI: 0.89, 1.13). Meanwhile, ozone’s effects on CHD were stronger with the increased duration of heatwave exposure (Figure 2D). The results above indicate that green space could attenuate the adverse effect of ozone on CHD, but heatwave exposure might worsen this adverse effect.

## 4. Discussion

Our study found evidence of higher CHD risk in infants whose mothers were exposed to ozone and heatwave during early pregnancy. Meanwhile, heatwaves could enhance ozone’s adverse impact on the CHD risk. On the contrary, the green space around the residence could lower the risk of CHD, and higher green space attenuated the adverse effect of ozone exposure on CHD.

Previous studies suggest that exposure to higher ozone concentrations during early pregnancy is associated with the risk of CHD [11]. A cohort study found that each 10 μg/m^3^ increase in ozone exposure in the first 4 weeks of gestation was associated with a 3% higher CHD risk [39]. Another population-based cohort reported that ozone exposure in the second month of gestation was positively associated with CHD (OR = 1.10, 95% CI: 1.03, 1.07) [40]. Our study also suggests that each interquartile range increases (31.48 μg/m^3^) in ozone exposure during 3 and 8 weeks after conception could increase CHD risk in offspring by about 7% (OR = 1.07, 95% CI: 1.02, 1.13). It should be noted that our study showed a U-shaped exposure–response curve association between ozone and CHD, which means a protective effect of ozone exposure under certain threshold. This indicates the complicated effect of ozone as a highly oxidizing gas [41]. Low concentrations of ozone have been reported to induce antioxidant responses in the body, reduce oxidative stress, and exert anti-inflammatory effects [42]. In addition, low ozone exposure may help maintain mitochondrial function, ensuring a stable cellular energy supply [43]. Moderate stimulation of reactive oxygen species (ROS) production at these low concentrations can also play a physiological role, as controlled ROS levels contribute to the regulation of the endometrial cycle, thereby supporting healthy embryo development [44]. On the contrary, once ozone concentrations reach the upper limit, it starts to induce oxidative stress and becomes a toxicant [45]. Collectively, these mechanisms provide a theoretical basis for the observed U-shaped relationship between ozone exposure and CHD. More studies are needed to reveal the dose‒effect relationship between ozone and CHD.

Although most studies reported positive associations between ozone and overall CHD, subtypes of CHD might react differently to ozone exposure. For example, studies suggested ozone was positively associated with VSD, TOF, and pulmonary stenosis (PS), but was negatively associated with ASD [40,46,47]. This study also observed inconsistent associations between ozone and CHD subtypes. For example, ozone exposure was significantly associated with VSD, AVSD, and PDA, but had no significant association with ASD. We speculated that CHD subtypes’ heterogeneity in development and etiology might lead to inconsistency in their reaction to ozone. Researchers proposed several potential biological mechanisms where ozone induces developmental abnormality in the heart. Ozone could produce superoxide, hydrogen peroxide, and hydroxyl radicals in the human body, cause oxidative stress, and affect cardiac development [48,49]. A cohort study conducted among young women showed that ozone exposure was associated with changes in the methylation levels of multiple inflammation-related gene sites, such as, TLR-2, ARG2, TPM4, C1QTNF1, and MIP, which play important role in fetal development [50]. Animal studies suggested that ozone exposure can disrupt the expression profile of microRNA (miRNA) which maintains cardiac metabolic homeostasis [51]. Meanwhile, ozone can cause embryonic lethality because of its strong oxidative activity, leading to abnormal fetal organ development [52].

Our study suggested that interquartile range increment of NDVI in 500 m, 1000 m, and 1500 m buffers was associated with a 5% to 6% lower risk of CHD (500 m buffer: OR = 0.93, 95% CI: 0.90, 0.97; 1000 m buffer: OR = 0.94, 95% CI: 0.90, 0.98; 1500 m buffer: OR = 0.93, 95% CI: 0.89, 0.97). A large population-based study also applied NDVI to estimate maternal green space exposure during the first trimester and found that each 0.1 increase in NDVI was associated with a 7% lower risk of CHD (OR = 0.93, 95% CI: 0.90, 0.96) [53]. Another large case–control study in China reported the reverse associations between NDVI in 500 m and 1000 m buffer and the risk of CHD (NDVI in 500 m buffer: OR = 0.95, 95% CI: 0.92, 0.98 per IQR increment; NDVI in 1000 m buffer: OR = 0.94, 95% CI: 0.91, 0.97 per IQR increment). Moreover, researchers also found that green space might reduce the concentrations of NO_2_, PM_1_, PM_2.5_, and PM_10_ to lower the risk of CHD, which is deemed a plausible explanation for the protective effect of green space on CHD [54]. Our study also indicated that green space exposure could attenuate the adverse impact of ozone on CHD, adding evidence of green space’s mitigating effect on air pollutants. Previous studies also indicate that higher residential greenness was associated with lower risks of maternal morbidities, including gestational diabetes mellitus and hypertension, which are risk factors for CHD [55,56,57,58]. Other potential mechanisms under the effect of green space include stress reduction and restoration, social interaction, and physical activity [59,60,61,62]. It should be noted that we only observed apparent beneficial effects among individuals with relatively high greenness levels, which reminded us that high-level residential greenness is an essential factor to be considered in urban construction.

Since early pregnancy is a critical period for cardiac development [63,64], most related studies focused on the associations between heatwave exposure and CHD in the first trimester. A retrospective study explored the association between extreme heat exposure and CHD, using daily maximum temperature exceeding the 90th and 95th percentile of its distribution during the warm season to define heat exposure. Researchers found extreme heat exposure in early pregnancy was positively associated with the risks of overall CHD (Prevalence ratios = 1.32, 95% CI: 1.24, 1.39). Meanwhile, this study also reports positive associations between heat exposure and ASD and PDA (ASD: PR = 1.87, 95% CI: 1.56, 2.25; PDA: PR = 2.83, 95% CI: 2.44, 3.29) [65]. Another case–control study using similar percentile thresholds reported that heat exposure during early pregnancy was significantly associated with VSD [29]. Auger et al. reported that exposure to temperatures exceeding 30 °C and lasting 15 days could increase the risk of ASD (PR = 1.37, 95% CI: 1.10, 1.70) [66]. Our results suggest that pregnant women from urban or high-income areas were more susceptible to heatwave exposure. In recent years, the rapidly increasing human activities have led to regional temperature imbalance. For example, less well-planned urbanization would result in a threatening phenomenon where urban areas experience warmer temperatures than the surrounding rural areas, also known as the heat island effect [67]. Therefore, pregnant women living in urban areas might be exposed to higher ambient temperatures than those in rural areas. Our study reveals the synergistic effects between heatwave exposure and ozone on CHD, consistent with the results reported by Jiang et al. [68]. That might be an important pathway where heat exposure induces heart defects. Limited green space and lower vegetation cover in urban settings also reduce natural cooling through evapotranspiration, further intensifying heatwave conditions. In contrast, rural areas typically benefit from greater vegetation cover and lower building density, which can mitigate extreme temperatures [69]. Such differences could also influence co-exposure patterns, for instance, higher urban temperatures can accelerate photochemical reactions that increase ground-level ozone formation, potentially compounding the adverse health effects during gestation [70]. Future studies should consider integrating urban–rural stratification in exposure assessment to capture these variations more accurately.

Moreover, previous studies have recognized several heat-related pathways. For example, the cardiac load of pregnant women increases significantly with the ambient temperature rising, and the need for blood redistribution reduces uterine blood flow, which may impede the normal development of the placenta and fetus [71]. In addition, high-temperature exposure can also trigger the heat shock response, involving release of heat shock protein, affecting fetal development [72,73].

Given that ambient ozone can infiltrate indoor environments and pose health risks, our findings emphasize the importance of ozone mitigation strategies. At the population level, these may include reducing precursor emissions (e.g., nitrogen oxides and volatile organic compounds) through stricter industrial and traffic regulations, promoting clean energy use, and improving urban ventilation. At the individual level, pregnant women could benefit from protective measures such as indoor air filtration and minimizing outdoor activities during high-ozone periods. Moreover, our study suggests that pregnant women may be particularly vulnerable under concurrent high temperature and elevated ozone concentrations, highlighting the urgent need for climate adaptation strategies that target maternal and fetal health. Potential approaches include integrated early warning systems combining heatwave and air pollution alerts, and urban planning interventions such as expanding green spaces, improving residential ventilation, and enhancing cooling infrastructures. Incorporating maternal health into broader climate resilience and public health policies will be critical for reducing the burden of adverse birth outcomes in a warming and increasingly polluted world. Our study explored the effect of ozone, green space, and heatwaves on CHD based on a surveillance system covering the entire province, which allowed our study to avoid recall bias to a certain extent. Limited studies have reported the combined effect of ozone, green space, and heatwave on CHD to date. Our study revealed the mitigating impact of green space on CHD and the synergistic effect between ozone and heatwave. However, some limitations in this study should be noted. First, the study population was selected from a CHD surveillance platform, and their information was collected in the routine clinical process. Therefore, information on some demographic factors related to CHD, such as maternal education, smoking and alcohol consumption, and physical exercise, cannot be obtained and controlled in our analyses. Second, different types of green space, such as grasslands, forests, and artificial landscapes, affect human health differently due to their physical and hydrological properties. However, due to the lack of information, we could not confirm the exact green space types with relatively stronger effects on CHD. Third, we only estimated the exposure around participants’ residences, not considering other places the pregnant women frequented or spent time, which might lead to exposure misclassification bias. Finally, our study population was selected from one province, and the results of our study may not be applicable to populations in other regions with different urban form, climate, and healthcare infrastructure. Future studies could address these limitations by collecting more comprehensive individual-level data, characterizing green space types more precisely, and incorporating personal mobility or multi-location exposure assessment to improve accuracy and generalizability.

## 5. Conclusions

Understanding the health impacts of ozone and heatwaves is crucial for promoting maternal and child health. In this study, we observed significantly elevated risks of neonatal CHD among pregnant women exposed to high levels of ozone and heatwaves, particularly during early gestation. Moreover, our analysis indicated that green space may mitigate the adverse effects of ozone, suggesting a potential protective role in CHD prevention. These findings strengthen the epidemiological evidence on environmental risk factors for congenital heart defects and underscore the importance of urban planning and environmental interventions. Further studies with larger populations and mechanistic investigations are warranted to confirm these associations and clarify underlying biological pathways.

## Figures and Tables

**Table 1:** Characteristics of the study population.

	Total (*n* = 27,236)	CHD Cases (*n* = 6809)	Controls (*n* = 20,427)	*p* Value
Maternal age (years, mean ± SD)	30 ± 5	30 ± 5	30 ± 5	0.616
Gestational weeks (weeks, mean ± SD)	38.29 ± 2.06	38.34 ± 2.04	38.27 ± 2.06	0.008
Infant sex (*n*, %)				0.501
boys	12,910 (47.40)	3203 (47.04)	9707 (47.52)	
girls	14,326 (52.60)	3606 (52.96)	10,720 (52.48)	
Birth weight (kilograms)	3.22 ± 0.62	3.25 ± 0.61	3.21 ± 0.62	<0.001
Parity (*n*, %)				<0.001
primipara	15,310 (56.21)	4497 (66.04)	10,813 (52.93)	
multipara	11,926 (43.79)	2312 (33.96)	9614 (47.07)	
Singleton pregnancy (*n*, %)	25,954 (95.29)	6601 (96.95)	19,353 (94.74)	<0.001
Conception seasons (*n*, %)				<0.001
spring	6086 (22.35)	1527 (22.43)	4559 (22.32)	
summer	7016 (25.76)	1570 (23.06)	5446 (26.66)	
fall	7151 (26.25)	1752 (25.73)	5399 (26.43)	
winter	6983 (25.64)	1960 (28.78)	5023 (24.59)	
Residential region (*n*, %)				<0.001
urban	18,700 (68.66)	4891 (71.83)	13,809 (67.60)	
rural	8536 (31.34)	1918 (28.17)	6618 (32.40)	
AYE (years)	9.10 ± 0.52	9.19 ± 0.58	9.07 ± 0.49	<0.001
Per capita disposable income (*n*, %)				<0.001
low-income	10,957 (40.23)	2461 (36.14)	8496 (41.59)	
high-income	16,279 (59.77)	4348 (63.86)	11,931 (58.41)	
O_3_ concentration (μg/m^3^, median (P_25_, P_75_))	106.47 (89.38, 120.86)	108.07 (87.88, 122.58)	105.97 (89.92, 120.48)	0.034
NDVI (median (P_25_, P_75_))				
500 m buffer	0.27 (0.21, 0.36)	0.27 (0.20, 0.35)	0.28 (0.21, 0.37)	<0.001
1000 m buffer	0.29 (0.23, 0.39)	0.28 (0.22, 0.38)	0.30 (0.23, 0.40)	<0.001
1500 m buffer	0.31 (0.24, 0.41)	0.29 (0.23, 0.40)	0.31 (0.24, 0.42)	<0.001
NOAA (*n*, %)				<0.001
no heatwave exposure	16,345 (60.01)	3829 (56.24)	12,516 (61.27)	
1 day	4607 (16.92)	1022 (15.01)	3585 (17.55)	
2 days	2744 (10.07)	886 (13.01)	1858 (9.10)	
3 days	3540 (13.00)	1072 (15.74)	2468 (12.08)	

AYE: average years of education per capita; NDVI: normalized difference vegetation index; NOAA_1 day: daily heat index exceeding the mean heat index within the warm seasons (1 May to 30 September) in the 5 years before conception by 10 degrees and lasting 1 day; NOAA_2 days: daily heat index exceeding the mean heat index 10 degrees and lasting 2 days; NOAA_3 days: daily heat index exceeding the mean heat index 10 degrees and lasting 3 days.

**Table 2:** Associations of maternal exposure with CHD.

	Model 1		Model 2		Model 3	
	OR (95% CI)	*p* Value	OR (95% CI)	*p* Value	OR (95% CI)	*p* Value
Ozone						
Q1	Ref.		Ref.		Ref.	
Q2	0.71 (0.65, 0.77)	<0.001	0.80 (0.74, 0.87)	<0.001	0.83 (0.76, 0.90)	<0.001
Q3	0.78 (0.72, 0.84)	<0.001	0.96 (0.87, 1.05)	0.367	0.96 (0.87, 1.05)	0.358
Q4	1.17 (1.08, 1.26)	<0.001	1.45 (1.32, 1.59)	<0.001	1.40 (1.28, 1.54)	<0.001
*p* for trend	<0.001		<0.001		<0.001	
per IQR increment	1.00 (0.95, 1.03)	0.718	1.11 (1.06, 1.17)	<0.001	1.07 (1.02, 1.13)	0.012
NDVI in 500 m buffer						
Q1	Ref.		Ref.		Ref.	
Q2	0.89 (0.82, 0.96)	0.002	0.89 (0.83, 0.97)	0.005	0.89 (0.82, 0.96)	0.003
Q3	0.88 (0.82, 0.95)	0.001	0.92 (0.84, 0.99)	0.032	0.92 (0.85, 1.00)	0.049
Q4	0.77 (0.71, 0.83)	<0.001	0.82 (0.75, 0.89)	<0.001	0.86 (0.79, 0.93)	<0.001
*p* for trend	<0.001		<0.001		0.002	
per IQR increment	0.89 (0.86, 0.92)	<0.001	0.91 (0.88, 0.95)	<0.001	0.93 (0.90, 0.97)	<0.001
NDVI in 1000 m buffer						
Q1	Ref.		Ref.		Ref.	
Q2	0.86 (0.80, 0.93)	<0.001	0.88 (0.82, 0.96)	0.002	0.88 (0.81, 0.96)	0.002
Q3	0.82 (0.76, 0.88)	<0.001	0.86 (0.79, 0.93)	<0.001	0.88 (0.81, 0.95)	0.002
Q4	0.75 (0.69, 0.81)	<0.001	0.81 (0.74, 0.88)	<0.001	0.86 (0.79, 0.94)	<0.001
*p* for trend	<0.001		<0.001		0.003	
per IQR increment	0.88 (0.85, 0.91)	<0.001	0.91 (0.88, 0.94)	<0.001	0.94 (0.90, 0.98)	0.002
NDVI in 1500 m buffer						
Q1	Ref.		Ref.		Ref.	
Q2	0.88 (0.82, 0.95)	0.001	0.91 (0.84, 0.99)	0.024	0.92 (0.85, 0.99)	0.031
Q3	0.76 (0.71, 0.83)	<0.001	0.81 (0.75, 0.88)	<0.001	0.83 (0.77, 0.91)	<0.001
Q4	0.75 (0.69, 0.81)	<0.001	0.81 (0.75, 0.88)	<0.001	0.87 (0.80, 0.96)	0.003
*p* for trend	<0.001		<0.001		<0.001	
per IQR increment	0.86 (0.83, 0.90)	<0.001	0.90 (0.86, 0.94)	<0.001	0.93 (0.89, 0.97)	0.001
NOAA						
no heatwave exposure	Ref.		Ref.		Ref.	
1 day	0.93 (0.86, 1.01)	0.078	0.95 (0.88, 1.03)	0.231	0.91 (0.84, 1.00)	0.053
2 days	1.56 (1.43, 1.70)	<0.001	1.48 (1.35, 1.62)	<0.001	1.31 (1.19, 1.44)	<0.001
3 days	1.42 (1.31, 1.54)	<0.001	1.36 (1.25, 1.48)	<0.001	1.29 (1.18, 1.40)	<0.001
*p* for trend	<0.001		<0.001		<0.001	

NDVI: normalized difference vegetation index; NOAA_1 day: daily heat index exceeding the mean heat index within the warm seasons (1 May to 30 September) in the 5 years before conception by 10 degrees and lasting 1 day; NOAA_2 days: daily heat index exceeding the mean heat index 10 degrees and lasting 2 days; NOAA_3 days: daily heat index exceeding the mean heat index 10 degrees and lasting 3 days; Model 1 adjusted for no covariate; Model 2 further adjusted for infant sex, mother’s age, birth weight, gestational weeks, conception seasons (spring, autumn, fall, and winter), parity (primipara or multipara), singleton or multiple pregnancy; Model 3 further adjusted for residential region (urban or rural), average years of education per capita, and per capita disposable income (low or high).

**Table 3:** Associations of maternal exposure with CHD subtypes.

	ASD		VSD		AVSD		PDA	
	OR (95% CI)	*p* Value	OR (95% CI)	*p* Value	OR (95% CI)	*p* Value	OR (95% CI)	*p* Value
Ozone (per IQR increment)	0.97 (0.91, 1.04)	0.440	1.20 (1.06, 1.36)	0.003	1.29 (1.15, 1.45)	<0.001	1.26 (1.17, 1.36)	<0.001
NDVI (per IQR increment)								
500 m buffer	0.90 (0.86, 0.95)	<0.001	0.96 (0.87, 1.04)	0.328	0.99 (0.91, 1.07)	0.758	0.93 (0.88, 0.98)	0.005
1000 m buffer	0.90 (0.86, 0.95)	<0.001	0.97 (0.88, 1.06)	0.484	0.98 (0.90, 1.06)	0.606	0.92 (0.87, 0.97)	0.006
1500 m buffer	0.90 (0.85, 0.95)	<0.001	0.97 (0.88, 1.07)	0.568	0.97 (0.89, 1.07)	0.565	0.91 (0.86, 0.97)	0.003
NOAA								
no heatwave exposure	Ref.		Ref.		Ref.		Ref.	
1 day	0.72 (0.64, 0.80)	<0.001	1.01 (0.84, 1.20)	0.951	1.28 (1.09, 1.51)	0.002	1.06 (0.95, 1.18)	0.274
2 days	1.39 (1.24, 1.56)	<0.001	0.95 (0.75, 1.19)	0.652	1.54 (1.27, 1.85)	<0.001	1.07 (0.94, 1.21)	0.329
3 days	1.27 (1.14, 1.42)	<0.001	1.29 (1.06, 1.56)	0.010	1.42 (1.18, 1.70)	<0.001	1.42 (1.24, 1.61)	<0.001

NDVI: normalized difference vegetation index; NOAA_1 day: daily heat index exceeding the mean heat index within the warm seasons (1 May to 30 September) in the 5 years before conception by 10 degrees and lasting 1 day; NOAA_2 days: daily heat index exceeding the mean heat index 10 degrees and lasting 2 days; NOAA_3 days: daily heat index exceeding the mean heat index 10 degrees and lasting 3 days; ASD: atrial septal defect; VSD: ventricular septal defect; AVSD: atrioventricular septal defect; PDA: patent ductus arteriosus; Each model adjusted for infant sex, mother’s age, birth weight, gestational weeks, conception seasons (spring, autumn, fall, and winter), parity (primipara or multipara), singleton or multiple pregnancy, residential region (urban or rural), average years of education per capita, and per capita disposable income (low or high).

## Data Availability

The data presented in this study are available on request from the corresponding authors due to privacy or ethical restrictions.

## Supplementary Materials

The following supporting information can be downloaded at: https://www.mdpi.com/article/10.3390/toxics13090716/s1, Figure S1. The selection of study population; Table S1. Sensitivity analyses for associations between maternal exposure and CHD. Table S2. Associations between maternal exposure and CHD stratified by infant sex. Table S3. Associations of maternal exposure with CHD stratified by maternal age. Table S4. Associations between maternal exposure and CHD stratified by residential region. Table S5. Associations between maternal exposure and CHD stratified by income.
