# Supplementary material for: Effects of Prenatal Exposure to Ozone, Heatwave and Green Space on Neonatal Congenital Heart Disease: A Case-Control Study in Eastern China
Source: Toxics. 2025 Aug 26;13(9):716. doi: 10.3390/toxics13090716 (PMC12473690; doi:10.3390/toxics13090716)
Supplement: Supplementary file 1 [file toxics-13-00716-s001.zip › toxics-3777037-supplementary.docx]

**Effects of prenatal exposure to ozone, green space and heatwaves on neonatal congenital heart disease: a case-control study in Eastern China**

Figure S1. The selection of study population

Table S1. Sensitivity analyses for associations between maternal exposure and CHD

Table S2. Associations between maternal exposure and CHD stratified by infant sex

Table S3. Associations of maternal exposure with CHD stratified by maternal age

Table S4. Associations between maternal exposure and CHD stratified by residential region

Table S5. Associations between maternal exposure and CHD stratified by income


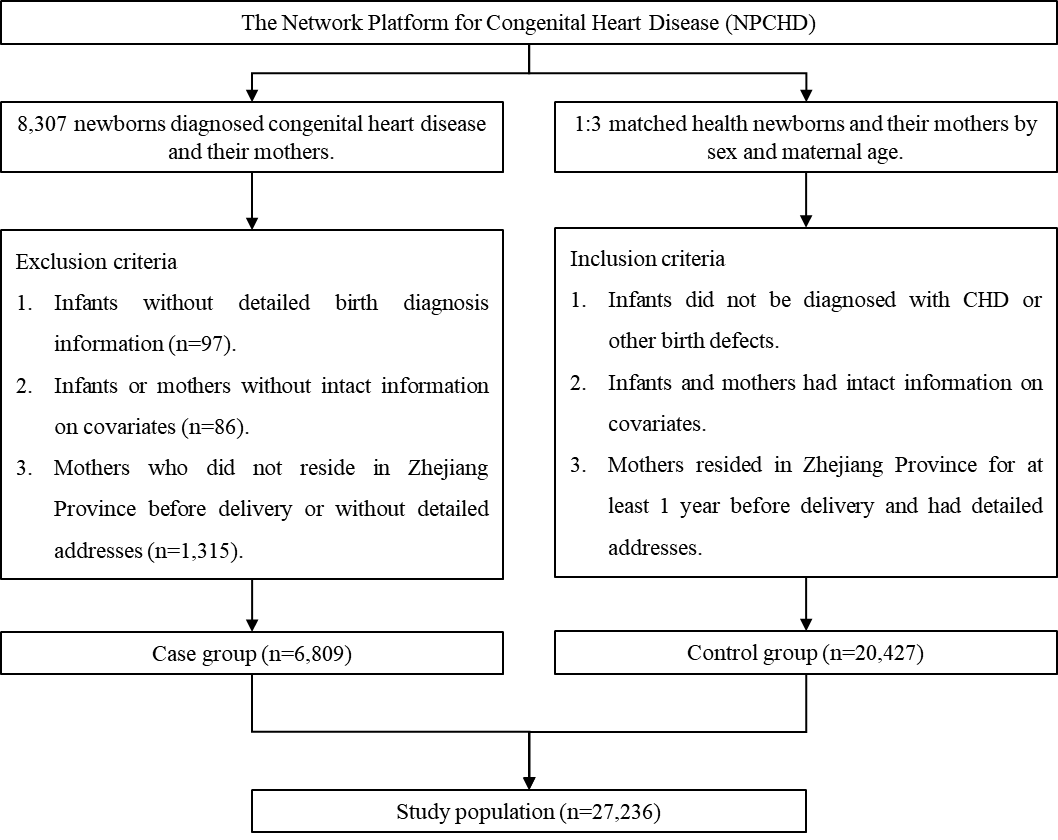


**Figure S1. The selection of study population.**

**Table S1. Sensitivity analyses for associations between maternal exposure and CHD**

|  | Excluding infants whose birth weight < 2.5kg^*^ | |  | Excluding mothers whose gestational week < 37 weeks^*^ | |  | Excluding multiple pregnancy^**^ | |
| --- | --- | --- | --- | --- | --- | --- | --- | --- |
|  | OR (95%CI) | *P* value |  | OR (95%CI) | *P* value |  | OR (95%CI) | *P* value |
| **Ozone** |  |  |  |  |  |  |  |  |
| Q1 | Ref. | |  | Ref. | |  | Ref. | |
| Q2 | **0.83 (0.76, 0.91)** | **<0.001** |  | **0.82 (0.75, 0.90)** | **<0.001** |  | **0.83 (0.76, 0.90)** | **<0.001** |
| Q3 | 0.97 (0.88, 1.07) | 0.494 |  | 0.95 (0.86, 1.06) | 0.367 |  | 0.97 (0.88, 1.07) | 0.572 |
| Q4 | **1.44 (1.30, 1.59)** | **<0.001** |  | **1.44 (1.30, 1.59)** | **<0.001** |  | **1.42 (1.29, 1.56)** | **<0.001** |
| *P* for trend | <0.001 | |  | <0.001 | |  | <0.001 | |
| per IQR increment | **1.07 (1.01, 1.13)** | **0.015** |  | **1.07 (1.01, 1.13)** | **0.021** |  | **1.07 (1.02, 1.13)** | **0.009** |
| **NDVI in 500m buffer** |  | |  |  | |  |  | |
| Q1 | Ref. | |  | Ref. | |  | Ref. | |
| Q2 | **0.90 (0.83, 0.97)** | **0.010** |  | **0.91 (0.84, 0.99)** | **0.029** |  | **0.86 (0.82, 0.96)** | **0.003** |
| Q3 | **0.91 (0.83, 0.99)** | **0.025** |  | **0.93 (0.85, 0.99)** | **0.019** |  | **0.91 (0.84, 0.99)** | **0.026** |
| Q4 | **0.85 (0.78, 0.93)** | **<0.001** |  | **0.84 (0.77, 0.93)** | **<0.001** |  | **0.86 (0.79, 0.94)** | **<0.001** |
| *P* for trend | 0.001 | |  | 0.008 | |  | 0.002 | |
| per IQR increment | **0.92 (0.89, 0.96)** | **<0.001** |  | **0.92 (0.89, 0.96)** | **<0.001** |  | **0.94 (0.90, 0.97)** | **<0.001** |
| **NDVI in 1,000m buffer** |  |  |  |  |  |  |  |  |
| Q1 | Ref. | |  | Ref. | |  | Ref. | |
| Q2 | **0.89 (0.82, 0.97)** | **0.005** |  | **0.90 (0.82, 0.98)** | **0.011** |  | **0.87 (0.81, 0.95)** | **0.001** |
| Q3 | **0.87 (0.80, 0.95)** | **0.002** |  | **0.88 (0.80, 0.96)** | **0.003** |  | **0.87 (0.80, 0.95)** | **0.001** |
| Q4 | **0.85 (0.78, 0.94)** | **<0.001** |  | **0.85 (0.77, 0.93)** | **<0.001** |  | **0.86 (0.80, 0.94)** | **0.001** |
| *P* for trend | 0.001 | |  | <0.001 | |  | 0.002 | |
| per IQR increment | **0.93 (0.89, 0.97)** | **<0.001** |  | **0.93 (0.89, 0.97)** | **<0.001** |  | **0.94 (0.90, 0.98)** | **0.003** |
| **NDVI in 1,500m buffer** |  |  |  |  |  |  |  |  |
| Q1 | Ref. | |  | Ref. | |  | Ref. | |
| Q2 | **0.90 (0.83, 0.98)** | **0.018** |  | **0.92 (0.84, 1.00)** | **0.042** |  | **0.91 (0.84, 0.99)** | **0.021** |
| Q3 | **0.83 (0.76, 0.91)** | **<0.001** |  | **0.84 (0.77, 0.92)** | **<0.001** |  | **0.82 (0.76, 0.90)** | **<0.001** |
| Q4 | **0.86 (0.78, 0.94)** | **0.001** |  | **0.85 (0.77, 0.94)** | **<0.001** |  | **0.87 (0.80, 0.95)** | **0.003** |
| *P* for trend | <0.001 | |  | <0.001 | |  | <0.001 | |
| per IQR increment | **0.93 (0.89, 0.97)** | **<0.001** |  | **0.93 (0.89, 0.97)** | **0.001** |  | **0.94 (0.90, 0.98)** | **0.002** |
| **NOAA** |  |  |  |  |  |  |  |  |
| no heatwave exposure | Ref. | |  | Ref. | |  | Ref. | |
| 1 day | 0.90 (0.84, 1.01) | 0.169 |  | 0.91 (0.86, 1.01) | 0.226 |  | 0.92 (0.83, 1.01) | 0.131 |
| 2 days | **1.32 (1.19, 1.47)** | **<0.001** |  | **1.33 (1.19, 1.47)** | **<0.001** |  | **1.32 (1.20, 1.47)** | **<0.001** |
| 3 days | **1.28 (1.16, 1.40)** | **<0.001** |  | **1.29 (1.17, 1.41)** | **<0.001** |  | **1.30 (1.18, 1.43)** | **<0.001** |
| *P* for trend | <0.001 | |  | <0.001 | |  | <0.001 | |

**NDVI**: normalized difference vegetation index; **NOAA_1day:** daily heat index exceeding the mean heat index within the warm seasons (May 1 to September 30) in the 5 years before conception by 10 degrees and lasting 1 day; **NOAA_2days:** daily heat index exceeding the mean heat index 10 degrees and lasting 2 days; **NOAA_3days:** daily heat index exceeding the mean heat index 10 degrees and lasting 3 days; *Each model adjusted for infant sex, mother’s age, birth weight, gestational weeks, conception seasons (spring, autumn, fall, and winter), parity (primipara or multipara), singleton or multiple pregnancy, residential region (urban or rural) and per capita disposable income (low or high); **Each model adjusted for infant sex, mother’s age, birth weight, gestational weeks, conception seasons (spring, autumn, fall, and winter), parity (primipara or multipara), residential region (urban or rural), average years of education per capita, and per capita disposable income (low or high).

**Table S2. Associations between maternal exposure and CHD stratified by infant sex**

|  | **Boys** | |  | **Girls** | | ***P* _for interaction_** |
| --- | --- | --- | --- | --- | --- | --- |
|  | OR (95% CI) | *P* value |  | OR (95% CI) | *P* value |  |
| **Ozone (per IQR increment)** | **1.10 (1.02, 1.19)** | **0.014** |  | 1.04 (0.96, 1.12) | 0.320 | 0.690 |
| **NDVI (per IQR increment)** |  |  |  |  |  |  |
| 500m buffer | **0.91 (0.86, 0.96)** | **<0.001** |  | 0.96 (0.91, 1.01) | 0.096 | 0.555 |
| 1,000m buffer | **0.91 (0.86, 0.97)** | **0.002** |  | 0.96 (0.91, 1.01) | 0.156 | 0.688 |
| 1,500m buffer | **0.90 (0.85, 0.96)** | **0.001** |  | 0.96 (0.91, 1.02) | 0.153 | 0.139 |
| **NOAA** |  | | | | | 0.294 |
| no heatwave exposure | Ref. | |  | Ref. | |  |
| 1 day | 0.92 (0.82, 1.04) | 0.175 |  | 0.90 (0.84, 1.05) | 0.056 |  |
| 2 days | **1.36 (1.18, 1.55)** | **<0.001** |  | **1.27 (1.12, 1.44)** | **<0.001** |  |
| 3 days | **1.35 (1.19,1.52)** | **<0.001** |  | **1.24 (1.10, 1.39)** | **<0.001** |  |

**NDVI**: normalized difference vegetation index; **NOAA_1day:** daily heat index exceeding the mean heat index within the warm seasons (May 1 to September 30) in the 5 years before conception by 10 degrees and lasting 1 day; **NOAA_2days:** daily heat index exceeding the mean heat index 10 degrees and lasting 2 days; **NOAA_3days:** daily heat index exceeding the mean heat index 10 degrees and lasting 3 days. Each model adjusted for maternal age, birth weight, gestational weeks, conception seasons (spring, autumn, fall, and winter), parity (primipara or multipara), singleton or multiple pregnancy, residential region (urban or rural), average years of education per capita, and per capita disposable income (low or high).

**Table S3. Associations of maternal exposure with CHD stratified by maternal age**

|  | **Mothers aged < 35 years** | |  | **Mothers aged ≥ 35 years** | | ***P* _for interaction_** |
| --- | --- | --- | --- | --- | --- | --- |
|  | OR (95% CI) | P value |  | OR (95% CI) | P value |  |
| **Ozone (per IQR increment)** | **1.06 (1.00, 1.12)** | **0.041** |  | 1.12 (0.98, 1.29) | 0.097 | 0.910 |
| **NDVI (per IQR increment)** |  |  |  |  |  |  |
| 500m buffer | **0.93 (0.90, 0.97)** | **0.002** |  | 0.93 (0.83, 1.02) | 0.139 | 0.731 |
| 1,000m buffer | **0.93 (0.89, 0.97)** | **<0.001** |  | 0.97 (0.87, 1.08) | 0.586 | 0.556 |
| 1,500m buffer | **0.92 (0.88, 0.97)** | **<0.001** |  | 0.97 (0.86, 1.09) | 0.629 | 0.462 |
| **NOAA** |  | | | | | 0.449 |
| no heatwave exposure | Ref. | |  | Ref. | |  |
| 1 day | **0.91 (0.83, 0.99)** | **0.033** |  | 0.91 (0.73, 1.14) | 0.420 |  |
| 2 days | **1.28 (1.16, 1.42)** | **<0.001** |  | **1.52 (1.15, 1.99)** | **0.003** |  |
| 3 days | **1.28 (1.17, 1.40)** | **<0.001** |  | **1.33 (1.03, 1.71)** | **0.025** |  |

**NDVI**: normalized difference vegetation index; **NOAA_1day:** daily heat index exceeding the mean heat index within the warm seasons (May 1 to September 30) in the 5 years before conception by 10 degrees and lasting 1 day; **NOAA_2days:** daily heat index exceeding the mean heat index 10 degrees and lasting 2 days; **NOAA_3days:** daily heat index exceeding the mean heat index 10 degrees and lasting 3 days. Each model adjusted for infant sex, maternal age, birth weight, gestational weeks, conception seasons (spring, autumn, fall, and winter), parity (primipara or multipara), singleton or multiple pregnancy, residential region (urban or rural), average years of education per capita, and per capita disposable income (low or high).

**Table S4. Associations between maternal exposure and CHD stratified by residential region**

|  | **Urban** | |  | **Rural** | | ***P* _for interaction_** |
| --- | --- | --- | --- | --- | --- | --- |
|  | OR (95% CI) | *P* value |  | OR (95% CI) | *P* value |  |
| **Ozone (per IQR increment)** | **1.07 (1.01, 1.14)** | **0.033** |  | 1.05 (0.96, 1.16) | 0.284 | 0.216 |
| **NDVI (per IQR increment)** |  |  |  |  |  |  |
| 500m buffer | **0.95 (0.91, 0.99)** | **0.012** |  | **0.90 (0.84, 0.98)** | **0.011** | 0.102 |
| 1,000m buffer | 0.96 (0.92, 1.00) | 0.079 |  | **0.89 (0.82, 0.96)** | **0.004** | **0.017** |
| 1,500m buffer | 0.96 (0.92, 1.01) | 0.091 |  | **0.87 (0.80, 0.95)** | **0.001** | **0.007** |
| **NOAA** |  | | | | | **0.017** |
| no heatwave exposure | Ref. | |  | Ref. | |  |
| 1 day | 0.94 (0.85, 1.03) | 0.179 |  | **0.83 (0.71, 0.97)** | **0.017** |  |
| 2 days | **1.39 (1.25, 1.55)** | **<0.001** |  | 1.09 (1.01, 1.46) | 0.364 |  |
| 3 days | **1.34 (1.21, 1.49)** | **<0.001** |  | 1.17 (1.08, 1.46) | 0.046 |  |

**NDVI**: normalized difference vegetation index; **NOAA_1day:** daily heat index exceeding the mean heat index within the warm seasons (May 1 to September 30) in the 5 years before conception by 10 degrees and lasting 1 day; **NOAA_2days:** daily heat index exceeding the mean heat index 10 degrees and lasting 2 days; **NOAA_3days:** daily heat index exceeding the mean heat index 10 degrees and lasting 3 days. Each model adjusted for infant sex, maternal age, birth weight, gestational weeks, conception seasons (spring, autumn, fall, and winter), parity (primipara or multipara), singleton or multiple pregnancy, average years of education per capita, and per capita disposable income (low or high).

**Table S5. Associations between maternal exposure and CHD stratified by income**

|  | **Low-income** | |  | **High-income** | | ***P* _for interaction_** |
| --- | --- | --- | --- | --- | --- | --- |
|  | OR (95% CI) | *P* value |  | OR (95% CI) | *P* value |  |
| **Ozone (per IQR increment)** | 1.01 (0.94, 1.09) | 0.708 |  | **1.11 (1.03, 1.20)** | **0.009** | 0.062 |
| **NDVI (per IQR increment)** |  |  |  |  |  |  |
| 500m buffer | 0.96 (0.91, 1.01) | 0.157 |  | **0.88 (0.83, 0.93)** | **<0.001** | 0.359 |
| 1,000m buffer | 0.95 (0.90, 1.01) | 0.101 |  | **0.92 (0.87, 0.97)** | **0.002** | 0.458 |
| 1,500m buffer | 0.96 (0.90, 1.01) | 0.138 |  | **0.91 (0.85, 0.96)** | **<0.001** | 0.166 |
| **NOAA** |  | | | | | **<0.001** |
| no heatwave exposure | Ref. | |  | Ref. | |  |
| 1 day | **0.89 (0.80, 0.98)** | **0.025** |  | 0.94 (0.83, 1.08) | 0.998 |  |
| 2 days | 1.11 (0.98, 1.27) | 0.111 |  | **1.70 (1.48, 1.94)** | **<0.001** |  |
| 3 days | 1.00 (0.90, 1.11) | 0.972 |  | **1.97 (1.70, 2.28)** | **<0.001** |  |

**NDVI**: normalized difference vegetation index; **NOAA_1day:** daily heat index exceeding the mean heat index within the warm seasons (May 1 to September 30) in the 5 years before conception by 10 degrees and lasting 1 day; **NOAA_2days:** daily heat index exceeding the mean heat index 10 degrees and lasting 2 days; **NOAA_3days:** daily heat index exceeding the mean heat index 10 degrees and lasting 3 days. Each model adjusted for maternal age, birth weight, gestational weeks, conception seasons (spring, autumn, fall, and winter), parity (primipara or multipara), singleton or multiple pregnancy, residential region (urban or rural), and average years of education per capita.
